# Munchausen by Internet: Current Research and Future Directions

**DOI:** 10.2196/jmir.2011

**Published:** 2012-08-22

**Authors:** Andy Pulman, Jacqui Taylor

**Affiliations:** ^1^School of Health & Social CareBournemouth UniversityBournemouthUnited Kingdom; ^2^School of Design, Engineering & ComputingPsychology Research CentreBournemouth UniversityBournemouthUnited Kingdom

**Keywords:** Munchausen by Internet, Internet trolls, identity deception, malingering, factitious disorder, deviance, social networking sites, health support groups

## Abstract

**Background:**

The Internet has revolutionized the health world, enabling self-diagnosis and online support to take place irrespective of time or location. Alongside the positive aspects for an individual’s health from making use of the Internet, debate has intensified on how the increasing use of Web technology might have a negative impact on patients, caregivers, and practitioners. One such negative health-related behavior is Munchausen by Internet.

**Objective:**

Munchausen by Internet occurs when medically well individuals fake recognized illnesses in virtual environments, such as online support groups. This paper focuses on the aspect of Munchausen by Internet in which individuals actively seek to disrupt groups for their own satisfaction, which has not yet been associated with the wider phenomena of Internet trolls (users who post with the intention of annoying someone or disrupting an online environment).

**Methods:**

A wide-ranging review was conducted to investigate the causes and impacts of online identity deception and Munchausen by Internet drawing on academic research and case studies reported online and in the media.

**Results:**

The limited research relating to motivation, opportunity, detection, effects, and consequences of Munchausen by Internet is highlighted and it is formally linked to aspects of trolling. Case studies are used to illustrate the phenomenon. What is particularly worrying is the ease with which the deception can be carried out online, the difficulty in detection, and the damaging impact and potential danger to isolated victims.

**Conclusions:**

We suggest ways to deal with Munchausen by Internet and provide advice for health group facilitators. We also propose that Munchausen by Internet and Munchausen by Internet trolling should be formally acknowledged in a revised version of the Diagnostic and Statistical Manual DSM-5. This will assist in effectively identifying and minimizing the growth of this behavior as more people seek reassurance and support about their health in the online environment. We also suggest directions for future research.

## Introduction

Lying to receive medical attention is not a new concept. Although it was not until the 1800s that factitious disorder was formally recognized, evidence of malingering dates back as far as Roman times. Munchausen syndrome was first described in 1951 [1], followed in 1977 by Munchausen syndrome by proxy [2]. More recently, the increasing use of the Internet to provide support for illnesses and other medical issues has introduced the concept of health-related online identity deception—Munchausen by Internet (identified in 2000) [3].

This paper reviews the research about the origins and evidence relating to these disorders. The limited research relating to motivation, opportunity, detection, effects, and consequences is highlighted with case studies. We conclude with practical and theoretical suggestions. We suggest practical ways for the health care community to deal with Munchausen by Internet and provide advice for health support group facilitators. We propose that Munchausen by Internet and Munchausen by Internet trolling be formally acknowledged and suggest future research directions.

### Types of Deception Regarding Health

#### Malingering

Malingering is defined as a deliberate behavior for a known external purpose [4]. Resnick [5] described three types of malingering: (1) *pure malingering *in which the individual falsifies all symptoms; (2) *partial malingering *in which an individual has symptoms but exaggerates the impact they have on daily life; and (3) *false imputation *in which the individual has valid symptoms but is dishonest as to the source of the problems. Other forms of malingering are *simulation *in which a person emulates symptoms of a specific disability and *dissimulation *in which the patient denies the existence of problems that would account for the symptoms (eg, drug abuse).

Historically, the Roman physician, Galen, presented the earliest evidence of malingering in the second century AD. One patient pretended to suffer from colic in order to avoid a public meeting and another faked a knee injury in order to remain home while his master took a long journey [6]. Causes of malingering vary. Although the malingering individual is seeking tangible gains, such as time away from work or avoiding an activity, the underlying motivation differs [3]. 

Malingering was widespread throughout Russia during the early 1950s because people sought to escape sanctions or coercion [7]. Russian physicians were limited by the state to only four medical dispensations. Patients were identified as: (1) needing medical care, (2) thinking they needed medical care, (3) faking, or (4) making direct pleas for medical dispensation. Low social trust is exhibited when certain ethnic groups have low trust in health care as an institution because of historical oppression and deception of their cultural group [8]. The early dependence upon doctors by poor Russian laborers has been said to have altered the doctor-patient relationship to one of mutual mistrust and deception [7]. If an individual patient trusts their doctor because the doctor has been assessed as trustworthy over time, this is an example of interpersonal trust. Recent health care literature has explored the role of patients’ trust in patient behaviors such as adhering to medical advice, malpractice litigation [9], and seeking health care services [10]. Some researchers believe that recent changes in health care practices are undermining the trust relationship between patients and physicians [11], with particular emphasis on the fact that technology is replacing the human element in medical practice thereby reducing patient’s trust in physicians [9]. Trust has been defined as a feeling (often based on inconclusive evidence) of certainty that a person or a thing will not fail [12]. There are several trust relationships. The three trust relationships relevant to this discussion are: (1) *interpersonal trust *defined as a human’s trust with another human whether face-to-face or through a device; (2) *social trust *defined as a human’s trust with a system or institution; and (3) *trust in automation *defined as a human’s trust with technology or a device.

There are no quantifiable numbers on how many people might misuse the Internet to abuse trust relationships, but many researchers have shown how the Internet lends itself to deception [3]. People might malinger online for external incentives, such as money or gifts, but fraud is usually the principle motivation [4]. Within the medical arena, the Internet offers anonymous access to vast amounts of information on illnesses and support groups for rare diseases, opening up opportunities for people with the urge to pretend they are sick and abuse trust [13].

#### Factitious Disorder and Munchausen Syndrome

Factitious disorder (FD) is an umbrella category covering a group of mental disturbances in which patients intentionally feign physical or mental illness without obvious benefit. Gavin [14] first described “factitious disease” in 1843. He described soldiers and seamen who mimicked illness to incite compassion or attention. The exact incidence of FD is unknown, but studies suggest that approximately 9% of hospitalized patients on specialty services in tertiary care have FD [15]. Modern study began in 1951 with an article by Asher [1], who coined the term “Munchausen syndrome” to describe a subtype of FD. The name came from a German baron who liked to embellish stories of his military exploits to impress listeners. Subsequently, lying and Munchausen were connected in German culture [16]. A series of patients whose medical histories consisted mainly of falsehoods and who visited and deceived hospitals and doctors have been described in the medical literature [1].

People with Munchausen syndrome go to incredible lengths to appear sick or to make themselves sick. For example, a young woman surreptitiously ingested laxatives to cause diarrhea, hypokalemia, and weight loss, and another young woman feigned cancer with the help of fabricated medical records in order to receive chemotherapy [17]. Those with Munchausen syndrome who have medical training are especially convincing. Others read up on diseases so they can mimic symptoms accurately [18]. Some become so proficient that they can fool doctors into ordering needless tests and even operations in some cases. Munchausen syndrome tends to be chronic and people with it usually become habitual deceivers [19]. Savino and Fordtran [17] proposed that it was likely that many cases of factitious cancer were never discovered and predicted that with advances in computer technology the quality of forged medical records would improve in the future. This is shown by the recent case of an individual using medical images obtained from the Internet to support claims of ankle dislocation [20].

#### Munchausen Syndrome by Proxy and Fabricated or Induced Illness

In 1977, Munchausen syndrome by proxy (MSP)—also known as factitious disorder by proxy—was first described by Meadow [2]. One mother had poisoned her toddler with excessive quantities of salt and another mother had introduced her own blood into her baby’s urine sample. MSP refers to a parent or other adult caretaker who repeatedly seeks medical attention for their children, whose symptoms they have faked or induced, sometimes causing real harm to the child, and/or subjecting them to unnecessary investigations and interventions. Many hypotheses have been proposed to explain MSP. Some have noted that patients with the condition often present traumatic events—particularly abuse and deprivation and numerous hospitalizations in childhood—and as adults may have lacked support from family and friends. Others consider that it allows patients to feel in control as they never felt in childhood [21]. Savino and Fordtran suggested that it might represent the patient’s attempt to cope with emotional distress [17].

In the United Kingdom, MSP is now termed “fabricated or induced illness” (FII) [22], although MSP is still widely used in other countries. The British Paediatric Surveillance Unit (BPSU) carried out a study of FII cases and identified 97 cases in the United Kingdom over a two-year period. This equated to 1 child in every 5000 being affected by FII, but it is likely that this figure underestimates the true scale of the problem. Another study estimates that the number of children affected by FII could be as high as 1 in 1100 [22].

#### Munchausen by Internet and Munchausen by Proxy by Internet

In 1983, the case of “Joan and Alex” shocked users of a CB radio channel of the national network, CompuServe, when a male psychologist (Alex) masqueraded as a disabled woman (Joan) in an attempt to use the trust and intimacy attained in the online interactions for his own social gain [23]. In 2000, Munchausen by Internet was identified by Feldman [3] to describe an individual seeking attention by playing out a series of dramatic near-fatal illnesses and recoveries that increasingly strain credulity. A Google search for the term yields more than 7000 search results. There is also an extensive Wikipedia entry [24] that has been revised 285 times since its creation in 2007—an average of 61.5 edits per year—suggesting that the term has now comfortably entered the online vernacular of Internet users. Munchausen by Internet can have devastating effects within online discussion groups, destroying trust when the hoax is exposed [3]. The virtual communities that were created to give support, as well as general non-medical communities, often express genuine sympathy and grief for the purported victims. However, when fabrications are suspected or confirmed the ensuing discussion can create schisms, destroying some communities and altering the trusting nature of members in others.

As yet, Munchausen by Internet has not been officially recognized by the American Psychiatric Association in the Diagnostic and Statistical Manual of Mental Disorders (DSM-IV). The DSM-5 is currently in review, but makes no mention of Munchausen by Internet although FD is listed in two proposed subtypes: (1) factitious disorder imposed on self and (2) factitious disorder imposed on another*. *However, these entries make no reference to the Internet in the diagnoses [25]. A generic search on the DSM-5 review website for the term “Internet” locates one relevant mention in illness anxiety disorder under somatic symptom disorders, but this does not mention FD. One of the diagnostic criteria is that the person “performs related excessive behaviors (eg, checking one’s body for signs of illness, repeatedly seeking information and reassurance from the Internet or other sources)...” [26]. The DSM-5 review proposes minor modifications to factitious disorders under the umbrella of somatic symptom disorders in their own chapter. The most important modification is the elimination of the distinction between factitious disorders involving predominantly physical versus psychological symptoms. Also, factitious disorder by proxy is now termed “factitious disorder imposed on another” [25], with the perpetrator receiving the diagnosis, not the victim.

New cases of Munchausen by Internet are identified regularly. Recent examples demonstrate the versatility of using online formats for FD, including the adoption of multiple personae and the substantial time and effort expended to contrive complex and dramatic fake identities and conditions [27]. One case documented a brother-sister dyad who created an elaborate narrative to lure a woman into providing time and attention under false pretences. Elizabeth, the victim, had multiple sclerosis and was seeking online support through a social networking website hoping to connect with others coping with chronic illness [27]. In another case, after being diagnosed with a chronic illness, Helen sought to better understand her rare condition by turning to Internet resources for more information [27]. Her research led her to discover an online support community and she joined a support group designed specifically for people with her disease. Helen created a number of fake personae: “Isabelle” (Helen’s good friend), “Justin” (her boyfriend), and Justin’s father and sister to carry on the story after “Justin” passed away. Helen also fabricated two other storylines including one that featured an ailing mother who had tragically lost two children to illness. Another case concerned a 44-year-old woman who said she had been diagnosed with chronic myeloid leukemia. Had the documents presented by her not aroused suspicion, she would have undergone a bone marrow aspirate and chemotherapy [28].

Munchausen by Internet has also expanded to MSP on the Internet [29]. In 2009, Emily McDonald was arrested for injuring her daughter, Dakota, who had been in and out of hospital since her premature birth. When Dakota did not recover in hospital and her blood cultures showed odd results, staff became suspicious and set up a camera in her hospital room. On video, they caught McDonald putting fecal matter into Dakota’s feeding tube. McDonald’s case appeared to be MSP; however, she was also posting about her daughter and her illness on her own blog (no longer publically accessible). Although not diagnosed with MSP, she admitted to second-degree injury to a child and was sentenced to 20 years in jail [30].

## Munchausen by Internet

### Negative Impact from Health-Related Online Support

Debate has intensified on how the increasing use of Web technology might have a negative impact on patients, caregivers, and practitioners. For example, there can be a high ratio of false or irrelevant information compared to useful information on the Web. Eysenbach et al [31] systematically reviewed studies of health website evaluations and found that the most frequently used quality criteria included accuracy, completeness, and technical criteria related to site design (eg, visual appeal, layout, and readability). In their review, the authors noted that in 70% of the studies they had examined, the quality of health-related Web content was found to be low according to the authors of the reviewed studies. The incidence of false data in online self-help groups is unknown, although assumed to be high because of the absence of group rules and guidelines and few controls to prevent people from posting erroneous or off-topic information. Joinson [32] noted that the format of a childcare email list, which seemingly encouraged venting and the name calling of parents and children amidst unconditional support of other caregivers, created an environment which led to the acceptance of practices that were not child-centered and were potentially damaging to the children. This was because the legitimization of negative attitudes and approaches could have led to the continuation of these behaviors. The online “pro-anorexia” underground is a movement that supports those with anorexia and adopts an anti-recovery perspective on the disease [33]. While encouraging a non-healthy diet to sustain an anorexic lifestyle, the movement also recommends the radical use of weight-loss pharmaceuticals—conventionally used to treat obesity—to pursue and maintain low body weight. There are similar movements in other online disease communities, such as supporters of chronic fatigue syndrome who advise abundant rest and avoidance of activity for sufferers, which is in direct opposition to medical advice [34]. Finally, the Internet may also play a major role in the development and spread of beliefs that are unsupported by scientific evidence. For example, the spread of information about Morgellons disease on the Internet has led to several cases of delusional parasitosis [35]. In response, Vila-Rodriguez and MacEwan [36] recommended in a letter to the American Journal of Psychiatry that an awareness of the capacity of the Internet to enable and spread shared delusional ideation was essential to current medical practice.

### Review of Recent Munchausen by Internet Literature

We conducted a review of Munchausen by Internet literature over the previous two years by using both academic and social media sources. Searches using the term “Munchausen by Internet” were conducted on both PubMed and Google Scholar. In 2012, there were 8 published articles listed on Google Scholar, but none were relevant because they either contained citations which referred to old research or had no specific link between Munchausen syndrome and the Internet. The Technorati search engine was used in addition to search the blogosphere for recent blog postings or blogs on the terms “Munchausen by Internet” and “Munchausen” generated during the first half of 2012, but no relevant blogs or posts were identified.

### Motivation

Because many instances of Munchausen by Internet take place in a group situation, social psychology offers a number of theories that can be applied to explain this type of online behavior. Drawing on disinhibition theory, Suler [37] highlighted two features of the Internet that made deception easier: (1) asynchronicity allows a dynamic approach to identity presentation and enables quick changes between identities and styles, and (2) the lack of feedback and the anonymity or unfamiliarity of the audience can reduce concern for others’ views. Similarly, Taylor and MacDonald [38] applied the theories of de-individuation and social identity to explain more uninhibited behavior and more self-disclosure in some online settings. Drawing on motivations for deception and group effects, Mealy et al [39] found that lies motivated by a desire to benefit others were considered to be more acceptable than lies that primarily benefited the self. Additionally, lying to the *out-group *(the social group to which someone does not identify) was perceived as being more acceptable than lying to the *in-group *(the social group to which someone psychologically identifies themselves with as a member).

Other areas of psychology offer theories of relevance, for example those relating to self-presentation and identity. Research has shown a link between low self-esteem and the need for popularity to the way individuals manipulate relationships and the way they perceive others’ online relationships [40]. Walther [41] noted there is a propensity for disinhibition, projection, and transference, wherein there are no visual or auditory inputs that can place the text in its proper context or assist the correct interpretation of that text. People fill in the missing pieces in the picture of others they meet online, not fully aware that the picture they are forming is based partly on their own unconscious desires regarding who they want that person to be and how they want them to act. This occurs at the same time as the person is taking advantage of the anonymity inherent in text-only communications to present their best possible face. A feedback loop can arise as these selective presentations are responded to in-kind, creating a hyperpersonal aspect to Internet communications. The Hyperpersonal Model [42] proposes that the Internet affects three parts of the communication process. These can be applied to understand the way in which Munchausen by Internet users manipulate the Internet to manage impressions and also to explain how others make interpersonal impressions based on that information. The three parts of the communication process are:

1. Receivers who have an idealized perception of the message sender because subtle context cues take on a stronger value in online communication. An absence of face-to-face cues means that receivers may be acutely sensitive to any subtle social or personality cues, so partners build impressions of one another based upon minimal cues.

2. Message senders have a greater opportunity to optimize their self-presentation to others and themselves.

3. The asynchronous channel allows more time for senders and receivers to consider the messages they send and receive, so that only using text can create an idealized picture.

It is possible that some Munchausen by Internet sufferers could be driven by the simple enjoyment of online deception as highlighted by a study of Web users that found that most online deceivers felt a sense of enjoyment while engaging in online deception [43]. In 1999, a columnist was introduced in the UK newspaper, *The Observer*, following his aborted suicide attempt and detailing the columnist’s last few months before he killed himself [44]. After protest, it was revealed that the column was a spoof. Chris Morris, an innovative but controversial British broadcaster, wrote the columns that highlighted the inherent cruel, dark comedy of fooling gullible members of the public with stories of fake medical conditions. This willingness of broadsheet media to embrace and portray a borderline style of comedy can have unpleasant outcomes within an Internet environment. One clue might lie in the power granted by online communities to quantify the sympathy for an illness or the shock of a death through comments boxes or replies to a journal thread [13]. During a lengthy battle against terminal illness, blog writers can attract support from thousands of friends who follow them through treatments and who become emotionally involved when they die. In more than one example, bereaved online friends have created tribute websites where they have posted poetry and photographs in memorial books. This can feed the desire of a narcissist and as they create an imaginary online long-term condition leading to a fake death, can provide a lonely individual with attention that they may never have previously known. This view is supported by Feldman [3], who linked the engaging and intense nature of these deceptions to sadism. This motivation can be seen to be at both intrapersonal (sadistic) and interpersonal (attention) levels.

Stokes [45] argued that online social networks offer more methods to manage the impressions of others than are available through structured websites. He referred to a study that found Facebook users’ identities were not the identities that they had established in the offline world, nor were they close to the identities that they had constructed in other anonymous online environments. They were the hoped-for possible identities users wanted to have in the offline world, but had not yet been able to establish.

There is little research regarding the psychology of Munchausen by Internet, but there are indications that some online self-presentation may be motivated by narcissistic or sadistic tendencies, as is the case with FD. In one analysis of patients with FD, it was found that 9 patients (50%) had borderline personality disorder, 6 (33%) had narcissistic personality disorder, and only 3 patients (17%) did not demonstrate coexisting self-pathology [15]. A study of Internet dating sites by Ellison et al [46] found that people acted differently in social networking environments depending on whether or not they were interacting anonymously. This finding has important implications for understanding identity in the online world because it indicates that online self-presentation varies according to the nature of the online setting. Oltmanns [47] described narcissism as a pervasive pattern of grandiosity, a need for admiration, and an exaggerated sense of self-importance. Mehdizadeh [48] associated the term with positive self-views of agentic traits, including intelligence, physical attractiveness, and power. Central to most theoretical models of narcissism, the use of social relationships is employed to regulate narcissistic esteem. However, narcissists do not focus on interpersonal intimacy, warmth, or other positive aspects of relational outcomes. Instead, they use relationships to appear popular and successful, and they seek attractive, high-status individuals as romantic partners [49]. Despite their tendency to seek out many superficial, empty relationships, narcissists rarely pursue these commitments for long periods of time. Relationships are pursued solely when an opportunity for public glory presents itself [49].

### Netiquette and Trolls

The aspect of Munchausen by Internet where individuals actively seek to disrupt and cause problems for their own satisfaction or enjoyment (sadism versus narcissism) has until now not been consciously associated with the wider phenomena of Internet “trolls.” We believe that this connection should be formally acknowledged to assist in controlling, effectively identifying, and minimizing the growth of this behavior as more people seek reassurance and support about their health in an online environment. Netiquette (short for “network etiquette”) is the dos and don’ts of online communication covering common courtesy online and the informal “rules of the road” of cyberspace [50]. A *troll* is someone who posts or sends messages online with the intention of annoying someone or disrupting a discussion or environment [51]. The practice of trolling has been compared to the fishing term in which a line is set in the water and the bait is dragged slowly back and forth in the hope of getting a bite [52].

Donath [52] outlined the ambiguity of identity in a disembodied virtual community and provided a concise overview of identity deception games, which trade on the confusion between physical and epistemic communities. Trolling has been portrayed disdainfully in mainstream media outlets, often referencing the willingness of some Internet trolls to go to extreme lengths in their attempts to elicit reactions. In 2010, the Australian government became involved after trolls defaced the Facebook tribute pages of two murdered children. The Australian Minister of Communications decried the attacks as evidence of the need for greater Internet regulation [53]. In the wake of these events, Facebook responded by strongly urging administrators to be aware of ways to ban users and remove inappropriate content from Facebook pages. It is recommended that ignoring a troll is almost always the best approach, because if nobody responds the troll will eventually get bored and go away [51]. Experienced participants in online forums know that responding tends to encourage trolls to continue disruptive postings—hence the oft-seen warning, “do not feed the troll” (DNFTT). However, experts tend to inhabit the tougher, streetwise environs of Internet technical or film forums rather than in the supportive and empathetic environment of an online health support group, which can cause more of a shock when Munchausen by Internet trolls are unmasked.

### Opportunity

Recupero [54] highlighted instances of psychiatric patients engaged in impression management to influence the outcomes of psychiatric interviews [55]. Impression management also plays an important role in online conversations. Barak [56] believes that many Internet users prefer others to perceive and interact with an online persona and that material chosen to post online can help to deepen a preferred social impression. Conversely, profiles posted on social networking sites (eg, Facebook) may contain information contradicting the evaluee’s intended impression. Photographs, perhaps artificially composed, and other material can be posted and tagged online showing a person’s name or identity without their knowledge or permission regardless of whether the person is familiar with or naïve about Internet influence. Similarly, records of negative behavior can remain online for years becoming part of an individual’s digital footprint [57].

Computers allow people with sufficient technical skills to access medical records and use them to falsify medical histories [20], while the open trusting environments of communication forums—established for the sole purpose of giving support to members facing significant health or psychological problems—are easily infiltrated because of the social nature of the groups. This is an endemic problem with online communication as the Internet helps to break down the physical barriers that assist in preventing the spread of lies [58]. The proliferation of newsgroups and chat rooms offers a limitless audience for fake narratives with people able to move from one support group to another [18]. Some, pretending to be ill, have joined more than one, and some might sign on to a single group multiple times by using different names and acting out different roles. A *sock puppet *is an online identity used for purposes of deception. The term, a reference to the manipulation of a hand puppet made from a sock, originally referred to a false identity assumed by a member of an Internet community who spoke to, or about, himself while pretending to be another person, but it now includes other uses of misleading online identities, such as those created to praise, defend, or support a third party [59]. The development of FD in online groups and forums is made easier by the anonymous and malleable nature of online identity along with easy access to the Internet, which allows sock puppets to thrive without any negative consequences to themselves. For example, Andrea, a 40-year-old single mother, began posting on an ovarian cancer forum that she had concerns over her worsening abdominal pain. Shortly after joining the forum, Andrea announced that she had been diagnosed with ovarian cancer. After being confronted by suspicious group members, Andrea confessed to her deception and the use of sock puppets. In addition to playing herself during her illness and online death, she had posed as her daughter “Brittney” and her daughter’s boyfriend “Chris” [27].

### Detection

Although some Munchausen by Internet perpetrators display a remarkable degree of research and endurance, able to stretch the fiction over many months, even the most dedicated can slip up eventually. Outwardly, there might not be any clues to suggest that they are anything other than normal support group members, but slight details can introduce contradictions and, although anyone caught up in the deception may be willing to forgive a slight oversight, some are more methodical. They are prepared to investigate so that few contradictions will escape over time. Savino and Fordtran [17] suggested that diagnosis of factitious cancer is usually made by detection of inconsistencies in medical history, by evidence of fabrication of medical records, by lies patients tell about their health insurance, or by doctors who begin to doubt the patient’s story. A story of prolonged survival with a usually lethal cancer has helped reveal factitious cancer in some cases. Based on his experience with Munchausen by Internet, Feldman [3] listed some methods of detection:

1. Posts consistently duplicating material in other posts, books, or health-related websites.

2. Characteristics of the supposed illness emerging as caricatures.

3. Near-fatal bouts of illness alternating with miraculous recoveries.

4. Fantastical claims, contradicted by subsequent posts, or flatly disproved.

5. Continual dramatic events in the person's life, especially when other group members have become the focus of attention.

6. Feigned blitheness about crises that will predictably attract immediate attention.

7. Others apparently posting on behalf of the individual having identical patterns of writing.

Griffiths et al [20] recommended that all clinicians question histories that did not match examination findings, ensuring that all radiographs were adequately labeled with patient-specific information, and being aware of radiographic inconsistencies.

There are several strategies commonly employed in confronting FD. In one case series, patients were carefully confronted with the factitious nature of their illness. Although 13 of the 33 (approximately 39%) admitted feigning illness, most of the patients’ illnesses improved following this strategic confrontation [15]. In most cases, group members’ discovery of Munchausen by Internet can lead to a similar strategic confrontation [3,27] with the typical response being a protest of innocence and an allegation of mistreatment by the group, followed by disappearance [3]. Due to the elusive nature of online identities, most wronged group members are unable to pursue the fakers. Many either lock their journals so that only their friends can access them, or else purge them entirely and deny the fraud. Suspicious group members are sometimes able to take screenshots as evidence of the fraud, but many perpetrators slip away, either sufficiently chastened to stop the deceptions or to simply reappear in another online group.

### Effects and Consequences

The Munchausen by Internet troll can be costly in several ways. A troll can disrupt the discussion on a newsgroup, disseminate bad advice, and damage the feeling of trust in a Web community. Furthermore, in a group that has become sensitive to trolling—where the rate of deception is high—some honest but naïve question can be quickly rejected as trolling. This can be off-putting to a new user who is immediately bombarded with accusations when venturing a first post. Even if an accusation is unfounded, being branded as a troll can be damaging to an online reputation. Herring et al [60] discussed the difficulty inherent in monitoring trolling and maintaining freedom of speech in online communities, concluding that inevitably harassment was more likely to occur in environments where lack of censure was a key factor. In wider discussion forums, the broadly accepted ethic of free speech may lead to tolerance of trolling behavior, further complicating the members’ efforts to maintain an open-yet-supportive discussion area, especially for sensitive topics. Reactions from Internet forums have been critical of media portrayals of trolls, stating that trolling is nothing new and has become part of accepted Internet culture. While not condoning the viciousness of troll attacks, forum discussions regularly express concern that mainstream media coverage of trolling ultimately results in more trolling because widespread attention represents the reaction that trolls seek. However, in the insular, empathic environment of a support group, the reaction can be more severe. Grady [18] detailed the case of a 15-year-old girl who communicated with members of a virtual support group for parents of babies who were critically ill. Kim claimed to have a baby requiring treatment and as she detailed the timeline of her baby’s treatment, other members of the group became personally involved and were devastated when she said her baby had died. Subsequently, she appeared online saying she was pregnant again and that she feared the second baby would be born prematurely. After the early birth of her second baby, the same cycle was reenacted, but this time a group member (a psychologist and the mother of twins born prematurely) gradually became suspicious. She confronted Kim, who subsequently posted a confession and apology. Kim withdrew from the group and was taken off the list by its owner, but her behavior had a negative effect on a group who had been trusting and close-knit until then [18]. Some parents expressed feelings of betrayal, and many stopped posting messages. People in the group agreed to provide information so that a coordinator could verify they were really parents of premature babies. Some new participants were put off by the atmosphere of suspicion, but the group gradually bounced back. However, those who encountered Kim would obviously never view new postings in quite the same way again.

Application of the term “troll” is highly subjective. Some readers may characterize a post as trolling, whereas others might regard the same post as a legitimate discussion contribution, even if controversial. Sometimes the term is used as a strategy to discredit an opposing position by attacking its proponent. Calling someone a troll makes assumptions about a writer’s motives. Regardless of the circumstances, controversial posts may attract a particularly strong response from those unfamiliar with the robust dialogue found in some online, rather than physical, communities. The popularity of Facebook means strangers can often build and maintain relationships entirely on the Internet. This anonymity permits any number of lies to be accepted as truth, and inventive deceptions, with whatever motive, can be carried all the way to an online grave. However, there is also the potential danger that overzealous group members might make the erroneous assumption that every death encountered online is fake. Most social network sites now allow relatives of deceased users to choose to keep profiles online as a memorial, allowing users to post tributes and messages, sometimes speaking of the dead in the third person, sometimes in the second person. In effect, a profile site is converted into a tribute site, a space of commemoration—a sort of open-ended electronic wake [45]. In the case of a real death, it can be just as harrowing for the bereaved to read comments claiming that the death has been faked.

## Conclusions

More research is required to be able to provide evidence-based advice to victims of suspected Munchausen by Internet trolls and for facilitators of discussion groups to effectively manage interactions. As this is one area of the literature that does not yet yield much information, one methodology that could possibly be adopted for further study is based on qualitative content analysis (QCA), which is gaining much support in studies of social interactions in online support communities [61]. QCA provides a way to study manifest and latent content within a body of text. Analysis of what the text says describes the visible components, referred to as the *manifest content*. Analysis of what the text talks about involves interpreting the underlying meaning of the text, referred to as the *latent content*. Therefore, manifest content might highlight descriptions of illnesses, while latent content could include descriptions of feelings of sufferers and the context of an illness. The manifest content is usually presented in categories, whereas the latent content is expressed as themes. QCA differs from pure qualitative research as it allows the researcher to emphasize differences between and similarities within codes and categories. Therefore, it could be used to differentiate types of Munchausen by Internet and its different motives.

When Munchausen by Internet seems likely, it might be practical to have some established group members gently question any dubious post owner privately. Although the typical response is vehement denial regardless of the strength of the evidence, the author typically will disappear from the group. In some Munchausen by Internet cases, much like FD cases, individuals can be both perpetrators and patients. For example, the previously described case of Helen who was diagnosed with a chronic illness and went on to create a number of fake personae [27]. Savino and Fordtran [17] suggested some useful steps for confronting patients suspected of FD:

1. Let the patient know what you suspect but without outright accusation.

2. Support the suspicion with facts.

3. Provide empathetic and face-saving comments.

4. Avoid probing to uncover the patient's underlying feelings and motivations so as to minimize disruption of emotional defences that are essential to her function.

5. Assure the patient that only those who need to know will be informed of the suspicion of factitious disease.

6. Make sure the staff demonstrate continued acceptance of the patient as a person worthy of their help.

7. Encourage psychiatric help, but if the patient resists do not force the issue.

However, once a perpetrator has been confronted, remaining members of the online group may need psychological help at an individual or group level depending on the extent of the deception and the health topic concerned. For example, individuals may need help in processing their feelings. As a group, help could be directed toward dealing with conflict and blame, and moving forward to refocus the group on its original goals with the aim of protecting and encouraging the original sense of trust. As Whitworth and de Moor [62] suggest, laws in a physical community are expressed in terms of physical actions and concrete objects that govern what people do, not what they think or feel. Historical law assumes a physical world constrained by time and space, but virtual environments have significantly different functionality. This means the virtual world is a functionally different world; it may not be appropriate, or even possible, to transfer laws from the physical to the electronic world. Therefore, laws must be re-invented by re-applying legitimacy concepts to virtual contexts such as formulating direct policy to protect health information users in the new world of Internet-based health searching and support. Legal sanctions have yielded some results in controlling the “acting out” of Munchausen’s syndrome, but the literature suggests that such measures are ineffective and can sometimes even reinforce bad behavior [63]. It is suggested that the best results within a physician-patient relationship can be achieved by approaching the dilemma from diverse angles [28].

Historically, the consequences for perpetrators have tended to be minor because few can be pursued or punished unless the wronged individuals are able to prove that the perpetrators have committed an illegal act. However, a 2005 legal case concerning self-help members pursuing an online campaign against a Munchausen by Internet member who challenged posts as defamatory suggests that wronged individuals are able to respond without fear of successful legal reprisal. They might also be able to win a precedent-setting civil case [64]. Indeed, there is a strong case for considering the sadistic misuse of health-related forums as a form of cybercrime, rather than as an everyday negative risk of using the Internet that must be tolerated and accepted. Consider if a malicious user deliberately (or accidentally) gave out medical information that resulted in a worsening of health or had fatal consequences. Internet protocol (IP) addresses of Munchausen by Internet trolls could be identified and Internet service providers (ISPs) could be enlisted to help identify and “out” frequent perpetrators such as been seen in recent online copyright disputes. Social network providers, such as Facebook, should tighten up their own procedures or, as an alternative, group users might want to consider relocating to more private group-based Internet communities, such as private Wikis [51]. Although these do not have the same large population of users, they might increase the security and lessen the chance of encountering a Munchausen by Internet troll online.

Enhanced self-regulation is the most positive action to reduce group risk. It might also be advisable for a health support group to identify a gatekeeper. Although adding extra layers of security and formality before a user could post might be viewed as onerous, the long-term benefits might be worth the additional effort. Facilitators could also clearly state to all members that although most people participating in support groups are honest, all members should balance their empathy with some degree of circumspection. Group members should be especially careful about basing any of their own health care decisions on uncorroborated information supplied in groups, just as they should with any other source on the Internet [65].

We have reviewed potential motivations and consequences for Munchausen by Internet behaviors, but it is clear that further research is necessary to investigate the psychology and methods of coping with Munchausen by Internet. However, there is a clear, compelling need to recognize that in addition to Munchausen by Internet being classed as a condition in its own right, there is a subset of people currently tagged as Munchausen by Internet sufferers who are actually Munchausen by Internet trolls purposefully harming well-intentioned support groups and abusing members for their own pleasure or enjoyment. We propose that Munchausen by Internet and Munchausen by Internet trolling be formally acknowledged in a revised version of DSM-5 (within the factitious disorder revisions), and that this sphere of behavior needs wider consideration and action, either by group users or by the creators of the host software. As Berners-Lee [66] said, “Technologists cannot simply leave the social and ethical questions to other people, because the technology directly affects these matters.”

## Abbreviations

BPSU: British Paediatric Surveillance Unit
DNFTT: Do not feed the troll
FD: factitious disorder
FII: fabricated or induced illness
IP: Internet protocol
ISP: Internet service provider
MSP: Munchausen syndrome by proxy
QCA: qualitative content analysis
